# Draft Genome Sequence of the Tacrolimus-Producing Bacterium *Streptomyces tsukubaensis* F601

**DOI:** 10.1128/genomeA.00385-17

**Published:** 2017-05-18

**Authors:** Gongli Zong, Chuanqing Zhong, Jiafang Fu, Ronghuo Qin, Guangxiang Cao

**Affiliations:** a Shandong Academy of Medical Sciences, Shandong Medicinal Biotechnology Centre, Jinan, China; b Shandong Jianzhu University, School of Municipal and Environmental Engineering, Jinan, China

## Abstract

*Streptomyces tsukubaensis* strain F601 was found to be a producer of the immunosuppressive drug tacrolimus. The draft genome sequence of this strain was approximately 8.52 Mbp. Genes involved in the biosynthesis of tacrolimus were identified in the genome. This draft genome sequence will provide insights into the genetic basis of tacrolimus biosynthesis and regulation.

## GENOME ANNOUNCEMENT

FK506 (tacrolimus) is a natural product with immunosuppressive and antifungal activities; it has been used clinically to prevent the rejection of transplanted organs and in the treatment of eczema (1, 2). Recently, clinical studies have shown numerous promising therapeutic properties of FK506, such as neuroprotection and regeneration (3, 4). FK506 was first detected in the fermentation broth of *Streptomyces tsukubaensis* 9993, which was isolated from a soil sample from Tsukuba, Ibaraki Prefecture, Japan, in 1984 (5). In the decades following its discovery, FK506 and its structural and functional analogs (such as FK520 and rapamycin) have been isolated from a variety of soil-dwelling *Streptomyces* species (6). FK506 biosynthesis involves a multifunctional polyketide synthase and nonribosomal peptide synthetase systems. The FK506 gene cluster has been partially or entirely sequenced from several streptomycete strains, such as *Streptomyces* sp. strain MA6548 (ATCC 53770), *Streptomyces* sp. strain KCTC 11604BP, *Streptomyces kanamyceticus* KCTC 9225, and *S. tsukubaensis* 18488 (6–9).

*Streptomyces tsukubaensis* F601 is a potent producer of FK506, and the original strain was isolated from forest soil in Shandong, China. Here, we report the whole-genome sequence of *S. tsukubaensis* F601 and its features.

The genome of strain F601 was sequenced using the Illumina HiSeq 4000 platform at the Beijing Genomics Institute (Shenzhen, China). The paired-end sequencing library was constructed with the NEBNext Ultra DNA library prep kit for Illumina (New England BioLabs, UK). Sequencing of the genome produced a raw data set of 10,766,446 paired-end reads. After removing low-quality reads, duplication reads, and adapter reads, about 1,500 Mb of clean data were obtained, which gave a 35.48-fold average coverage of the genome. The clean reads were assembled *de novo* with SPAdes (10) and then polished with SSPACE-Standard and GapFiller to obtain scaffold sequences (11, 12). Using SPAdes, a total of 8,506,753 bp of genomic sequence was assembled, which covers about 95.01% of the predicted genome; the G+C content was calculated to be 70.45%.

The genome was annotated using the Prokaryotic Genome Annotation Pipeline (PGAP) version 3.2 software on NCBI. Additional functional annotation was performed using the RASTtk server (13). The automated gene prediction identified 7,257 coding genes (CDSs) and 73 RNA-coding genes, including 64 tRNA, two 5S rRNA, one 16S rRNA, one 23S rRNA, and five noncoding RNA genes. The genome also harbors the FK506 biosynthetic gene cluster, which partially shares high sequence similarity with the FK506 gene clusters from *S. kanamyceticus* KCTC 9225 and *S. tsukubaensis* 18488. This draft genome sequence will help us further understand the molecular mechanisms of FK506 biosynthesis and regulation.

### Accession number(s).

The draft sequence of *S. tsukubaensis* strain F601 obtained in this whole-genome shotgun sequencing project has been deposited in DDBJ/ENA/GenBank under accession no. MVFC00000000. The version described in this paper has accession no. MVFC01000000.
